# A New Microörganism Discovered in Pork

**Published:** 1895-05

**Authors:** Frank J. Thornbury

**Affiliations:** Buffalo, N. Y.


### A NEW MICROÖRGANISM DISCOVERED IN PORK.

By FRANK J. THORNBURY, M. D., Buffalo, N. Y.

In my work as microscopist in the Bureau of Animal Industry, I have commonly observed in various parts of the muscular system of swine, undergoing inspection with reference to the presence of trichine, a peculiar fungus. This fungus presented itself in the form of bundles of threads which have various colors and are intermingled with the muscle fibers, or found separate in a clump under the field of the microscope.

Out of 1,000 hogs inspected daily at the government abattoir, Buffalo, I have found fifty, on an average, to be infected by this organism.

The parts of the carcass from which samples are taken for the trichinous inspection are the diaphragm, neck and loin respectively, hence these were the parts in which I have usually found the fungus. As corroborated by Prof. Miller, of Berlin, this organism belongs to the saccharomyces or yeast group. It has very distinctive morphological characteristics and I here present for your inspection pure cultures in every media which we have at our disposal. At a later time I will detail the peculiarities of growth of this organism in the various media and give the results of my experiments upon animals. The former are particularly interesting and present contrast in many respects to any organism with which we have thus far had to deal. This can only be a brief outline of my contribution. I direct your notice to drawings of different culture tubes made by Mr. F. C. Busch. They are very accurate and serve to give you an impression of the luxuriant manner in which the organism thrives in artificial media, and of the coloration which it produces. I will later show you photo-micrographs, produced by Dr. Hill, and will have their duplicates thrown upon the screen for more detailed inspection. A pathogenic potency of this fungus —saccharomyces porcus—is shown by the destruction of white mice and rats twenty-four hours after inoculation. I have recovered the organism from the blood of these animals, which is found to be heavily laden.

My paper will appear in full in the official reports of the Bureau of Animal Industry, United States Department of Agriculture.

---

The Massachusetts Board of Registration in Medicine received during 1894, 4,390 applications for licenses; 2,817 graduates applied and 200 who had practiced for three years; 294 applicants were rejected.—*Chicago Medical Recorder.*

